# GNPAT promotes immunosuppression in hepatocellular carcinoma by activating the plasmalogen-PPARγ pathway to drive M2 macrophage polarization

**DOI:** 10.3389/fimmu.2026.1765930

**Published:** 2026-02-24

**Authors:** Meng Hu, Nan Zhang, Ya-Qi Wang, Xiao-Ming Wang, Yun Shi, Min Yao, Lian-Guo Hou, Ling-Ling Jiang

**Affiliations:** 1 Ministry of Education Key Laboratory of Neural and Vascular Biologys, Department of Biochemistry and Molecular Biology, Hebei Medical University, Shijiazhuang, Hebei, China; 2 Department of Complex Preparation, Shijiazhuang No. 4 Pharmaceutical, Shijiazhuang, Hebei, China; 3 College of Integrative Chinese and Western Medicine, Hebei University of Chinese Medicine, Shijiazhuang, Hebei, China; 4 Department of Clinical Laboratory, Hebei Province Hospital of Chinese Medicine, Shijiazhuang, Hebei, China; 5 Department of Clinical Laboratory, The First Hospital of Tsinghua University, Beijing, China

**Keywords:** biomarkers, drug sensitivity prediction, GNPAT, hepatocellular carcinoma, immune cell infiltration, peroxisome

## Abstract

Hepatocellular carcinoma (HCC) is a major threat to human health worldwide. Its suboptimal responses to current therapies are largely attributable to the immunosuppressive tumor microenvironment (TME) that dampens the efficiency of available treatments. Although metabolic reprogramming is regarded as a hallmark of HCC, the exact role of peroxisomal metabolism in immune evasion is poorly understood. By integrating bioinformatic analysis of TCGA-LIHC datasets and peroxisomal gene profiling, glyceronephosphate O-acyltransferase (GNPAT) was identified as a regulator of HCC pathogenesis. GNPAT was highly expressed in malignant tissues and positively associated with poor clinical outcomes and immunosuppressive cellular infiltration types. Functional experiments showed that GNPAT facilitated the proliferation, migration, and resistance to apoptosis of HCC cells in an autocrine manner via enhancing plasmalogen synthesis and downstream PPAR pathway activation. Interestingly, overexpression of GNPAT in HCC cells polarized macrophages to the M2-like phenotype and reinforced immunosuppressive TME through the plasmalogen-PPAR axis. An unrecognized mode of immunometabolic crosstalk mediated by peroxisomal metabolism in HCC was thereby revealed, providing a preclinical rationale and mechanistic basis for future exploration of GNPAT inhibition as a potential therapeutic strategy to antagonize immunosuppression and enhance antitumor immunity.

## Graphical Abstract

**Figure:**
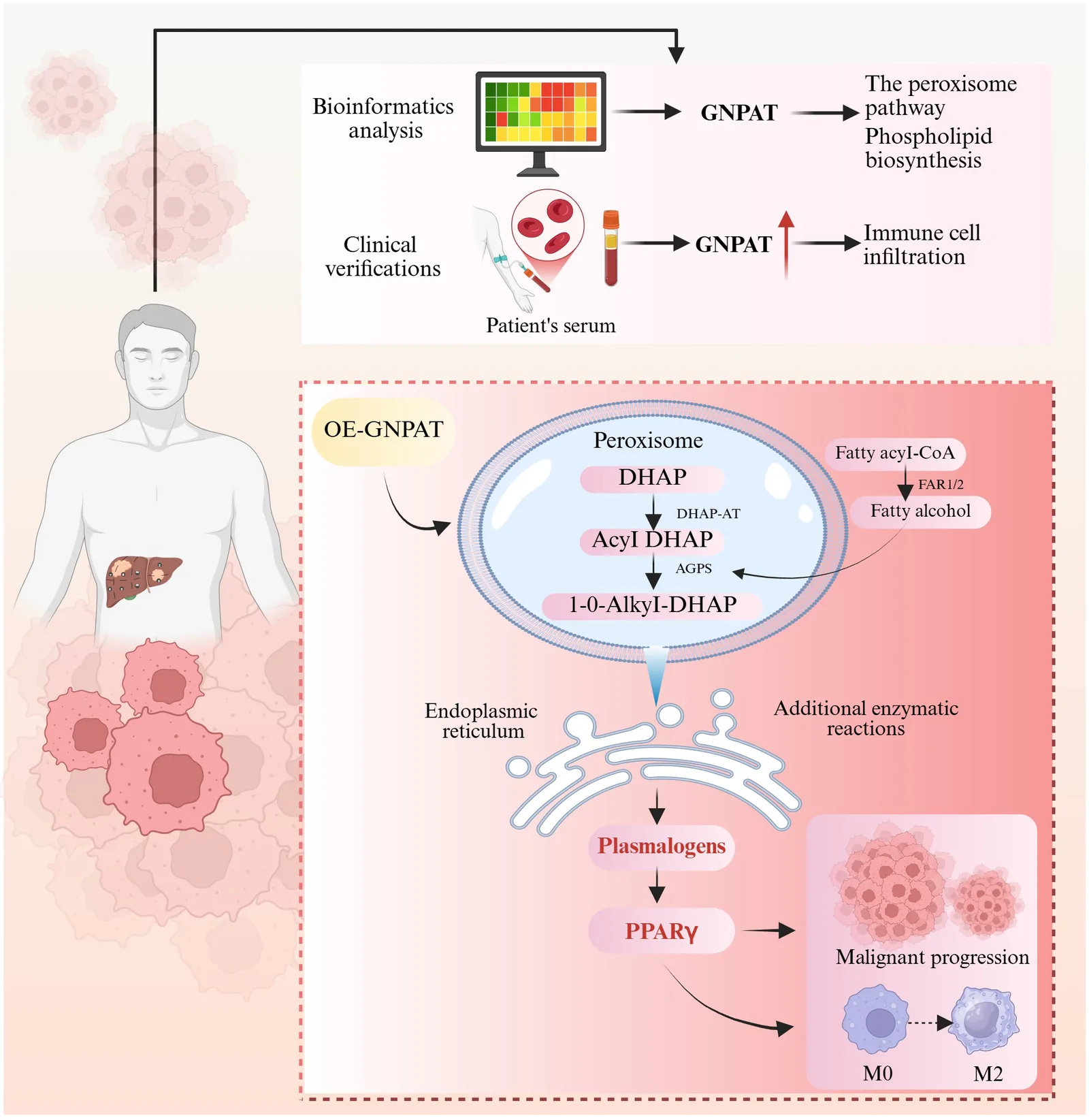
Schematic diagram illustrating that GNPAT promotes HCC progression and remodeling of the immune microenvironment through the regulation of the plasmalogen–PPARγ axis. Graphical Abstract was created by biorender and licensed to be published.

## 1 Introduction

Hepatocellular carcinoma (HCC) is a malignancy of significant pathogenic complexity, which often forms on the background of chronic liver disease and disrupted metabolic homeostasis (1–5). Peroxisomes are essential organelles responsible for fatty acid β-oxidation, lipid synthesis, and reactive oxygen species (ROS) scavenging, playing crucial roles in the preservation of metabolic and redox balance. There is an increasing body of evidence showing that defective peroxisomal conditions exist in the pathogenesis of HCC by disturbing lipid homeostasis, increasing oxidative stress, and degrading cellular integrity (6–9). Through outliers in peroxisomal gene expression, similar to metabolic defects, and tumor progression, the relevance of the outlined genes in the study of liver pathobiology has been emphasized (10–12).

Important studies also evidence that peroxisomal impairment facilitates the progression of hepatocarcinogenesis via interrelationship mechanisms of lipid imbalance, oxidative stress, and perturbation of redox signaling (13–15). The PPAR family is one of the transcriptional regulators involved in transcriptional regulation linked to peroxisomal activities that act as the mean of controlling peroxisomal metabolism of fatty acids oxidation and ROS/RNS (16). The relationships between the peroxisomal metabolic pathways and the nuclear receptor signaling, especially the PPAR-mediated transduction represent a key dimension in cancer biology. The peroxisome-derived ligand-activated nuclear receptor Peroxisome proliferator-activated receptor gamma (PPARγ) is a lipid-sensitive receptor, which is involved in the processes of lipid metabolism, inflammatory responses, and the immune system in general (17–19). It is interesting to note that some lipid species generated in peroxisomes such as the unsaturated fatty acids and specialized pro-resolving mediators are also endogenous PPARγ agonists to provide a direct molecular linkage between peroxisomal lipid metabolism and immunomodulatory signaling (11, 20, 21). This connection proposes that the peroxisomes could modify the tumor microenvironment (TME) by generating lipid mediators that impact the immune cell behavior, such as macrophage polarization status. However, the mechanisms of action by which peroxisomes mediate immune responses in HCC are not understood.

Recent studies have revealed a complex orchestration of peroxisomal changes in HCC that would be associated with both impairment of functions as well as metabolic reconfiguration. Under common conditions, conventional peroxisomal functions, including β-oxidation capacity and the ability to prevent ROS, often are reduced in tumor tissues, which is evidenced by reduced expression of such critical enzymes as catalase (22, 23). Conversely, there are biosynthetic pathways that are paradoxically improved including plasmalogens. Ether lipid biosynthesis, in its turn, has become a selectively enriched peroxisomal functioning in HCC, which signifies tumor-specific metabolic adjustments that facilitate cancer development (24, 25). Importantly, this metabolic re-modeling seems to be not restricted to autonomous metabolism of tumor cells since the peroxisoma abnormalities are being identified with an ability to alter the immune landscape (13). Modern discoveries also indicate cell-type-specific peroxisomal changes, and unique expression manifestations among the malignant hepatocytes, stromal elements, and immune cellular groups in the HCC ecosystem (16, 26, 27). All these data suggest that, instead of general dysfunction throughout peroxisomes, selective reprogramming of these subcellular structures occurs and contributes to metabolic and immunomodulation adaptation in HCC.

On this basis it was postulated that essential regulatory pathways in HCC, intertwined between metabolic reprogramming and immune suppression, are constituted by the critical peroxisome associative genes, particularly those that regulate the synthesis of ether-based phospholipids. There is still a substantial knowledge gap regarding the specific peroxisomal enzymes that drive this process and the way they contribute to the metabolic products that promote an immunosuppressive TME. In this regard, to answer these questions, a combined bioinformatics analysis was performed to find and confirm essential peroxisome-related genes in HCC. Our study specifically aimed to delineate how the primary candidate gene coordinates plasmalogen metabolism and PPARγ signaling to concurrently enhance tumor cell aggressiveness and promote M2-like macrophage polarization. These results provide a mechanistic insight into peroxisome-based immunometabolic reprogramming in HCC demonstrating novel possibilities of immune restoration with therapies.

## 2 Materials and methods

### 2.1 Data collection

The Liver HCC dataset was downloaded from the UCSC Xena platform (https://xenabrowser.net). Sample selection from TCGA database employed these inclusion criteria: (1) histologically confirmed primary solid tumor specimens with matched adjacent non-tumor tissues; (2) transcriptomic profiling data derived from frozen patient samples. The final cohort comprised 369 tumor specimens and 50 matched normal controls. Additionally, a reference set of 79 peroxisome-associated genes was obtained from the KEGG database (https://www.kegg.jp). All datasets were processed under uniform inclusion criteria to ensure comparability across samples.

### 2.2 Identification of peroxisome-related differentially expressed genes

The bioinformatic analyses for biomarker discovery and validation were performed in accordance with the REMARK guidelines. DEGs between hepatocellular carcinoma and adjacent normal tissues were identified using the edgeR package in R. Intersection of these DEGs with the peroxisome-related gene set obtained from KEGG yielded a subset of pDEGs.

### 2.3 Machine learning for biomarker screening

Prognostically significant pDEGs were identified through univariate and multivariate Cox proportional hazards regression implemented with R survival package. Resultant hazard ratios were visualized using forest plots. For refined variable selection, LASSO regression was applied with optimal penalty parameters determined via 10-fold cross-validation. To evaluate the predictive efficacy of selected pDEGs, TCGA survival records were curated by removing duplicate entries and randomly partitioning patients into training and validation sets at a 1:1 ratio. Time-dependent ROC analysis was conducted using survival ROC software, where larger AUC values were interpreted as enhanced predictive accuracy.

### 2.4 Functional enrichment and protein-protein interaction analysis

PPI networks were generated through the GeneMANIA web resource (http://genemania.org/). Functional annotation of gene sets was performed via Gene Ontology (GO) and KEGG pathway enrichment analyses using the ClusterProfiler package in R, with a statistical significance threshold set at P < 0.05.

### 2.5 Correlation between biomarker expression and clinical factors

Samples were stratified into high- and low-expression subgroups according to median Glyceronephosphate O-acyltransferase (GNPAT) mRNA levels. Associations between GNPAT expression and clinicopathological staging were visualized through boxplots generated with the ggpubr package in R.

### 2.6 Immune infiltration analysis

Applying the ESTIMATE algorithm that provides the abundance of stromal and immune cells using transcriptomic profiling was used to characterizeTME composition in HCC specimens. The identification of key genes made the threads split into high- and low-expression groups using median values of expression to compare the stromal and immune scores. Gene expression signature-immune infiltration level correlation patterns were evaluated by applying the ESTIMATE pack in R. Moreover, relative fractions of 22 types of immune cells were deconvoluted using CIBERSORT computational method (https://cibersortx.stanford.edu/). Once the samples of HCC were partitioned into two expression-based clusters, the comparative distributions of populations of immune cells were represented in the form of violin plots created in the ggplot2 package in R.Together, these analyses enabled integrated quantification of stromal/immune content and immune cell composition across GNPAT-defined subgroups.

### 2.7 Prediction of immunotherapy response

Tumour Immune Dysfunction and Exclusion(TIDE) computational model was used to determine possible reaction of HCCs to immune checkpoint blockade therapy. This is a methodology that measures tumor immune evasion based on a dysfunctional or exclusion mechanism of T cells which presents an estimate of likely sensitivity to checkpoint blockade. Further, Subclass Mapping was done to compare the progress of transcriptomics in the high and low-expression subgroups of GNPAT to reference samples of reported immunotherapy responders. This comparative method evaluated the presence of expression signatures in the individual subgroups that were found to be molecularly homologous to the signatures of the presence of the responses to either CTLA-4 [anti-cytotoxic T-lymphocyte-associated antigen 4] or PD-1 [anti-programmed cell death protein 1] therapeutic antibodies.

### 2.8 Drug sensitivity prediction

The data available in the Genomics of Drug Sensitivity in Cancer resource was subjected to systematic analysis of chemosensitivity relationships by comparing the expressions of GNPAT and half-maximal inhibitory concentrations (IC50) of a variety of conventional chemotherapeutic agents. R pRRophetic algorithm was used to make drug sensitivity predictions on all specimens that were using transcriptional profiles to predict therapeutic response. There was a comparative evaluation of the estimated IC50 values between subgroups of GNPAT high- and low-expression groups. Differential drug sensitivity was statistically evaluated by the Wilcoxon rank-sum test, providing a non-parametric assessment of differences between expression-based cohorts.

### 2.9 Lipidomics analysis

The analysis of lipid in biological samples was done using an optimized version of the Bligh-Dyer method, which enables the recovery of the largest amount of lipid in the organic layer and conservation of the integrity of volatile metabolic compounds. After extraction, the dried lipids film was resuspended in the isotope-labeled internal standard solution according to the next quantification and normalization required in the consequent analysis. A comprehensive screening of lipidomics in the Exion UPLC system was improved with a QTRAP 6500Plus mass spectrometer (Sciex, USA) together with electrospray ionization to provide a high sensitivity level and specific detection of the molecular forms. The polar lipids were chromatographically separated using the Phenomenex Luna silica column (150 x 2.0 mm, 3 m) with binary mobile phase system, mobile phase A (comprised of chloroform/methanol/ammonia 89.5: 10:0.5) and mobile phase B (comprised of chloroform/methanol/ammonia/water 55:39: 0.5: 5.5). Multiple reaction monitoring (MRM) was used to identify and determine lipid species with good accuracy in relative quantification and unequivocal ion discrimination.

### 2.10 Transmission electron microscopy analysis

In order to perform ultrastructural analysis, the 5×10^7^ cells of hepatocellular carcinoma were fixed, dehydrated, and embedded following a 3-step procedure involving an acetone gradient at ambient temperature. Thin slices (around 1μm) were stained with uranyl acetate then stained with lead citrate to increase the visibility of peroxisomes. TEM was used to perform imaging and random microscopic fields were chosen to be systematically evaluated with regard to peroxisomal morphology.

### 2.11 RT-qPCR analysis

Paired tumor and adjacent non-tumorous liver tissues were obtained from HCC patients. Total RNA was isolated from all specimens following a standardized extraction protocol to preserve RNA integrity for downstream applications. Purified RNA was subsequently converted to complementary DNA using a commercial reverse transcription system (Toyobo, Japan). Gene expression quantification was performed via RT-qPCR, with relative transcript levels determined by the 2^−ΔΔCt method using endogenous reference genes for normalization. Primer sequences validated for target gene amplification are comprehensively detailed in **Table 1**.

**Table 1:** Primer sequence used for mRNA RT-qPCR.

Gene	Sequences (5′−3′)
PPARγ	F: ACACGATGCTGGCGTCCTTGATG R: TGGCTCCATGAAGTCACCAAAGG
ACOX1	F: CCTCTGGATCTTCACTTGG R: TGGGTTTCAGGGTCATACG
CD36	F: AACCCAGAGGAAGTGGCAAAG R: AAGTGCATCATCGTTGTTCATACA
β-actin	F: GACAGTGAAGGCTCAAAGATGG R: AGAGGTCTTTACGGATGTCAACGT

### 2.12 Immunohistochemical staining

Tissue sections were blocked with goat serum prior to incubation with a primary antibody targeting GNPAT. Following extensive washing, specimens were sequentially treated with a biotin-conjugated secondary antibody and horseradish peroxidase-streptavidin complex. Diaminobenzidine (DAB) served as the chromogenic substrate for visualization, with hematoxylin providing nuclear counterstaining. Stained sections were examined using a Leica microscope, and relative GNPAT protein abundance was quantified through staining intensity analysis performed with HistoQuest software.

### 2.13 Cell culture and grouping

The human HCC cell line HepG2 (ATCC, USA) was maintained in DMEM (HyClone, USA) supplemented with 10% FBS (HyClone, USA). To investigate GNPAT’s functional role in plasmalogen biosynthesis, cells were allocated into three experimental conditions: control, GNPAT-knockdown (si-GNPAT), and GNPAT-overexpression (OE-GNPAT) groups. For mechanistic studies examining plasmalogen-mediated tumor proliferation via PPARγ signaling, four treatment conditions were implemented: control, OE-GNPAT, OE-GNPAT with PPARγ antagonist T0070907 (OE-GNPAT+T007, 10 µM, MedChemExpress, USA), and si-GNPAT with hexadecyl plasmalogen supplementation (si-GNPAT+C16 P, using 10 µM C16:0 plasmalogen, Avanti Polar Lipids, USA).

Macrophage polarization assays utilized a Transwell co-culture platform, where M0 macrophages were co-cultured with HepG2 cells transfected with either GNPAT-overexpression constructs (OE-HepG2) or control vectors (NC-HepG2). Experimental configurations included: OE-HepG2+M0 (co-culture with GNPAT-overexpressing cells), OE-HepG2+M0+T007 (co-culture under PPARγ inhibition), and M0+C16 P (macrophages exposed to C16:0 plasmalogen alone). All cell lines underwent authentication through short tandem repeat profiling before experimental use.

### 2.14 Separation, purification, and purity identification of pPE

Plasmenylethanolamine (pPE) isolation and structural characterization followed previously established protocols (28). Total lipid extracts were prepared, followed by phospholipid enrichment through cold acetone precipitation and subsequent silica gel column chromatography. Further purification of plasmalogen and ether phospholipid species was achieved using large-scale silica gel chromatography (280 × 800 mm). Structural identification of purified fractions involved LC-MS analysis, with compound verification based on retention behavior, accurate mass measurements, and characteristic tandem mass spectral fragmentation. Sample purity was assessed by comparing chromatographic profiles of isolated plasmalogens against a phosphatidylethanolamine (PE 14:0/14:0) reference standard using liquid chromatography-mass spectrometry.

### 2.15 Immunofluorescence microscopy assays

For immunofluorescence studies, cells were plated on coverslips or in 24-well plates and exposed to various treatments. Fixation and permeabilization conditions were optimized according to subcellular localization of target antigens. Detection of Arg1 and ABCD3 employed fixation with 2% paraformaldehyde in PBS following the membrane permeabilization in 0.1% Triton X-100 (20 min at room temperature). The two-step protocol also facilitated antibody penetration. Following aldehyde quenching with 100 mM ammonium chloride for 10 min, the cells were labeled for PPARγ with pre-chilled acetone/methanol (1:1) for 20 min.

Following permeabilization, nonspecific binding sites were blocked by incubating the sections with 10% normal goat serum for 1 h. Primary antibody incubation proceeded overnight at 4 °C to ensure specific antigen recognition. After thorough washing, specimens were exposed to fluorophore-conjugated secondary antibodies (FITC or Alexa Fluor derivatives) for one hour under dark conditions. Nuclear counterstaining utilized DAPI (30 minutes) when required. Mounting was performed with 20% glycerol or Vectashield H1000 medium to preserve fluorescence integrity. Specificity controls included PBS-treated specimens, with final imaging conducted using fluorescence or confocal microscopy.

### 2.16 EdU assay

HepG2 cells were plated in 96-well plates (1×10^4^ cells per well) to ensure uniform distribution and attachment. Following a 24-hour incubation under standard culture conditions to establish monolayer integrity, cells were pulsed with 100 µL of 50 µM EdU for 2 hours to label replicating DNA. Cellular architecture and maintenance of nucleic acid integrity was ensured by subsequent fixation in 4% paraformaldehyde (20 minutes room temperature). The procedure used to detect incorporated EdU was as follows: the Cell-Light™ EdU Apollo^®^ 488 Imaging Kit (RiboBio, China) using DAPI, to visualize nucleus, to perform incorporation of EdU detecting plastic flow into nucleus. Fluorescence microscopic analysis was done after final washing procedures and indicated the successful detection of EdU positive nuclei, which indicated proliferating cell populations.

### 2.17 Transwell assay

The assays were Transwell chamber assays which were used to measure the cell migration and invasion ability of the cells through porous membranes. For migration assays, serum-free medium containing 5×10^4^ cells was added to the upper chamber, and the bottom chamber was loaded with 20% FBS medium, which served as a chemoattractant. Invasion assays were conducted using Matrigel-coated membranes (BD Biosciences) to replicate physiological barriers, where these coated membranes were incubated 4for 4 h at 37°C before cell seeding. After a 24-hour incubation, the cells that had transmigrated to the lower surface of the membrane were fixed with 4% paraformaldehyde and then stained with crystal violet. Microscopic quantification of stained cells was used as a quantitative analysis of migratory and invasive potential.

### 2.18 Western blotting analysis

Protein extraction from cultured cells utilized RIPA buffer (Thermo Fisher Scientific, China) following established protocols to ensure complete solubilization of cellular proteins. Protein concentrations were detected colorimetrically by a bicinchoninic acid (BCA) assay kit. For immunoblotting, equal protein aliquots (50 μg) underwent electrophoretic separation on 10% SDS-polyacrylamide gels, followed by transfer to PVDF membranes. Membranes were blocked with 5% skim milk in TBST for 90 minutes at 25°C before overnight incubation at 4°C with primary antibodies from Proteintech: GNPAT (14931-1-AP; 1:1,000), E-cadherin (20874-1-AP; 1:10,000), N-cadherin (22018-1-AP; 1:2,000), Vimentin (10366-1-AP; 1:20,000), and PPARγ (16643-1-AP; 1:1,000). After washing, membranes were probed with HRP-conjugated secondary antibodies (CW0103/CW0110S; CWBio; 1:1,000) for 2 hours. Protein bands were visualized via enhanced chemiluminescence (Thermo Fisher, USA) and quantified using ImageJ software, with β-actin serving as loading control for normalization.

### 2.19 ELISA analysis

Cytokine concentrations (TGF-β, IL-10, VEGF) in culture supernatants were quantified with commercial ELISA kits (JRDUN Biotechnology, China). Absorbance readings were normalized to total protein content determined by the Pierce BCA Protein Assay (Thermo Scientific, USA), with final results expressed as nanograms per gram of total protein.

### 2.20 Flow cytometry

CD206 surface expression on co-cultured macrophages was quantified through flow cytometric analysis, with untreated M0 macrophages serving as negative controls. Following detachment using cell scrapers and resuspension in chilled PBS with 2% FBS, cells were pretreated with human Fc receptor blocking reagent to minimize nonspecific antibody binding. For specific detection of the M2 polarization marker CD206, cells were stained with PE-conjugated anti-human CD206 monoclonal antibody for 30 minutes at 4°C under light-protected conditions. After thorough washing to remove unbound antibodies, samples were analyzed on a BD FACSCanto II system (BD Biosciences, USA), enabling precise quantification of CD206-positive populations based on fluorescence emission profiles.

### 2.21 Statistical analysis

R (v4.3.1) was used to carry out all statistical calculations and data representation, which is a complete environment in the application of analytical processes. Enrichment analysis p-values were adjusted using BenjaminiHochberg adjustment in order to control the detection of false findings. Wilcoxon rank-sum test was used as a non-parametric distribution comparison tests as a group comparison. Further statistical analysis and visualization were done using GraphPad Prism 9 (GraphPad Software). To demonstrate the quantitative data of 3 or more independent replicates, the result is represented as mean with standard deviation. Two-group comparisons were using Student t-tests (independent or paired design), and multi-group comparisons were performed using the one-way ANOVA with *post-hoc* tests of Dunnett. The analysis of the flow cytometry data and the population quantification was done using the FlowJo software (Tree Star). Statistical significance was defined as p < 0.05.

## 3 Results

### 3.1 Integrated bioinformatics analysis identifies GNPAT as a prognostic biomarker in HCC

**Figure 1:**
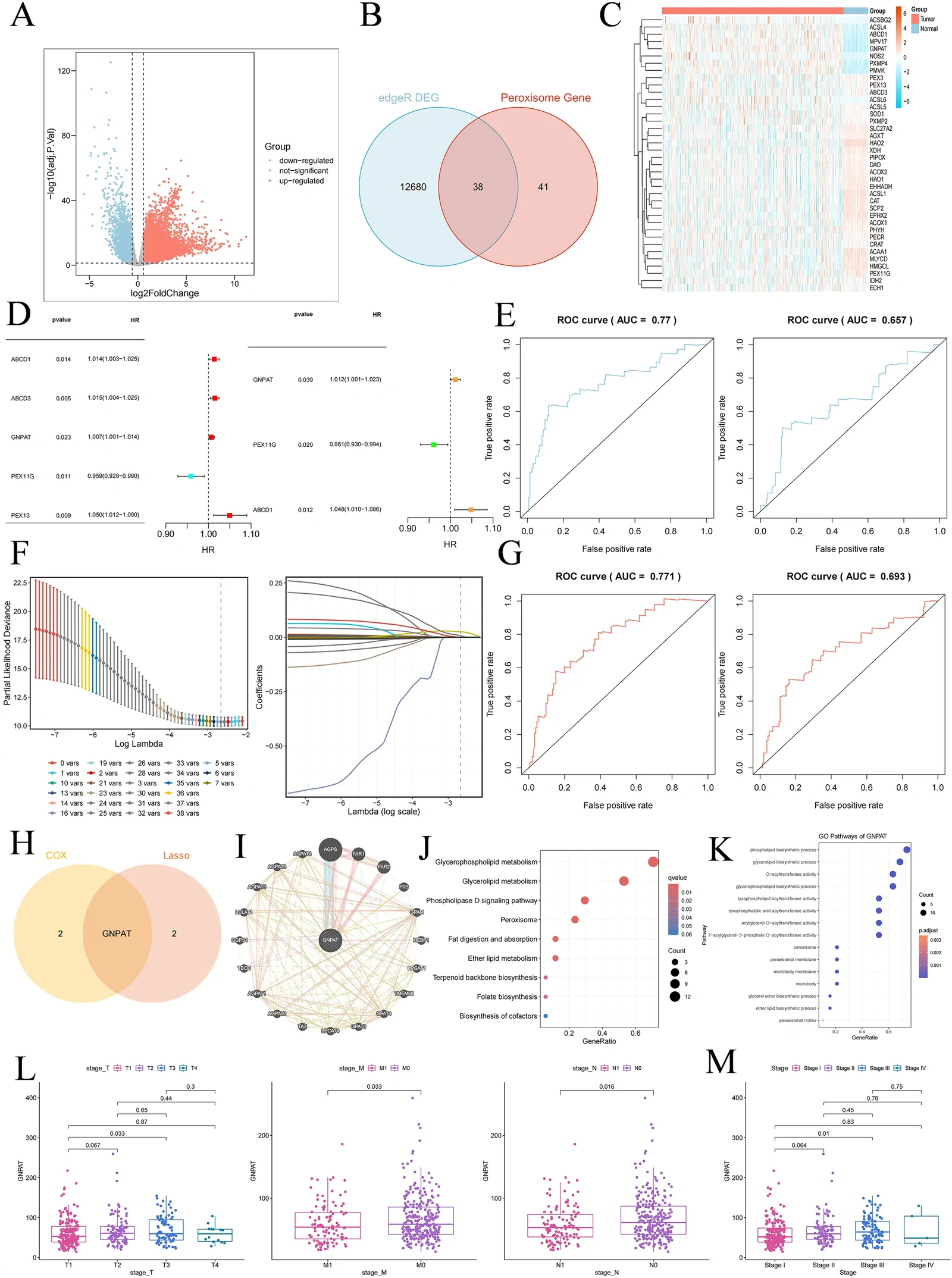
Identification of GNPAT as a key peroxisome-related biomarker in HCC through bioinformatics analysis. **(A)** Volcano plot of DEGs in HCC from the TCGA cohort. **(B)** Venn diagram identifying 38 pDEGs by intersecting DEGs with a peroxisome gene set. **(C)** Heatmap of the 38 pDEGs expression patterns in tumor and normal samples. **(D)** Cox regression analysis of the 38 pDEGs. **(E)** ROC curve assessing the prognostic performance of the Cox model. **(F)** LASSO regression analysis with 10-fold cross-validation for feature selection. **(G)** ROC curve assessing the prognostic performance of the LASSO model. **(H)** Venn diagram integrating Cox and LASSO results to identify GNPAT as a final candidate biomarker. **(I)** PPI network of GNPAT. **(J)** KEGG pathway enrichment analysis within the PPI network. **(K)** GO functional enrichment analysis within the PPI network. **(L)** Boxplot of GNPAT expression across different TNM stages. **(M)** Boxplot of GNPAT expression across tumor stage I/II/III/IV.

In-depth review of the TCGA-LIHC cohort showed that there were 12, 718 differentially expressed genes in hepatocellular carcinoma which included 9, 746 up-regulated and 2, 972 down-regulated transcripts (**Figure 1A**). Intersection of the peroxisome-associated gene set with the DEGs revealed 38 pDEGs, the expression patterns of which were decomposed using hierarchical clustering (**Figures 1B, C**). GNPAT was observed to be the most important prognostic factor for overall survival by univariate Cox and LASSO regression analyses (**Figures 1D, F, H**). The predictive model was shown to be very valid with values above 0.6 on the ROC analysis (**Figures 1E, G**). The network analysis of protein-protein interaction showed that GNPAT is associated with the key lipid-metabolic enzymes, such as AGPS, FAR1, and PTS (**Figure 1I**). GNPAT was functionally enriched and associated with peroxisomal pathways, glycerophospholipid metabolism and ether lipid biosynthesis (**Figures 1J, K**). Clinical validation demonstrated that GNPAT increased progressively as the TNM stage advanced with a peak of TNM stage III (**Figures 1L, M**).

### 3.2 GNPAT expression associates with immune microenvironment remodeling and therapeutic sensitivity

**Figure 2:**
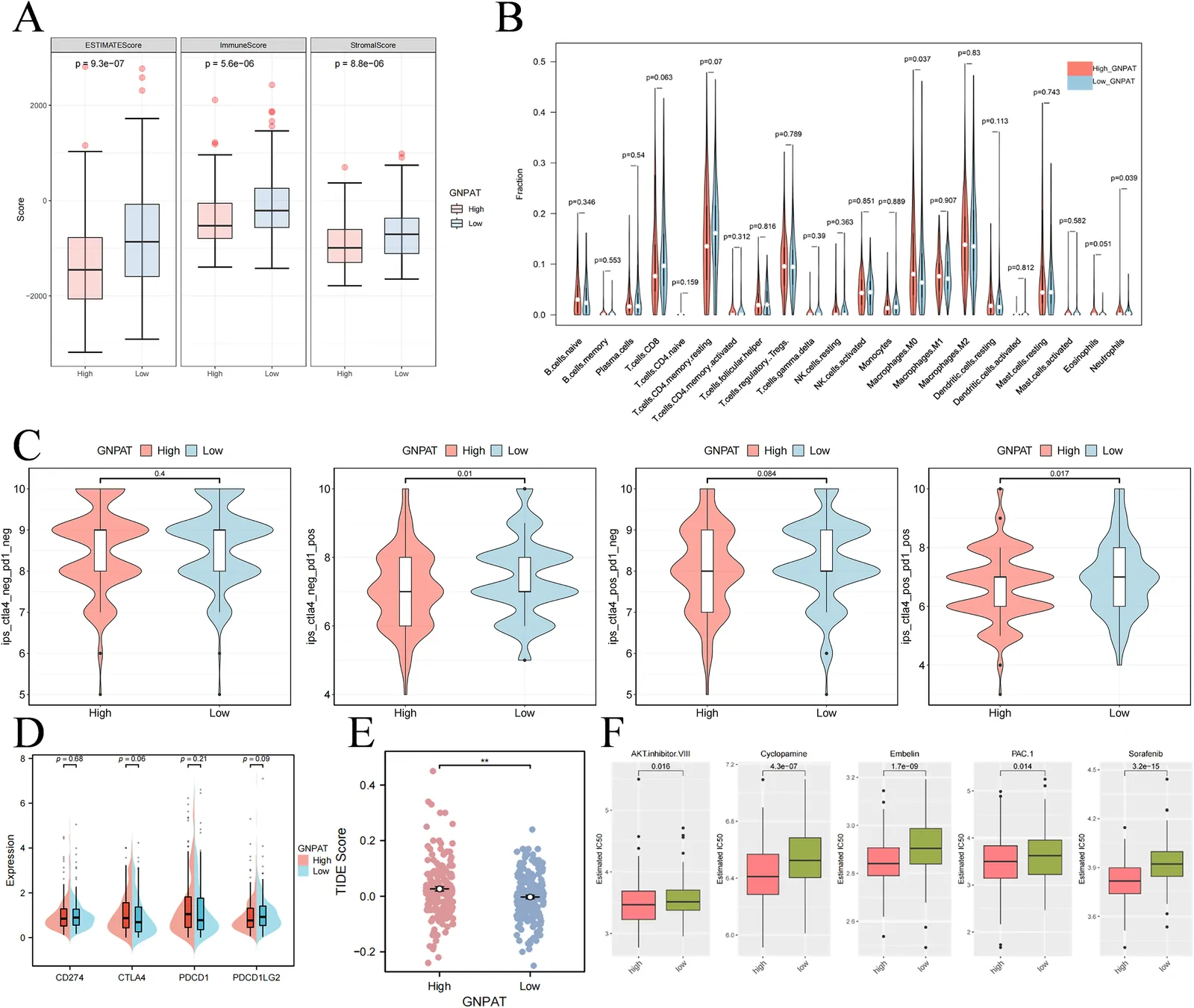
GNPAT expression correlates with altered immune infiltration and drug sensitivity. **(A)** Boxplot of the immunological score stratified by high and low GNPAT expression. **(B)** Violin plot showing the landscape of tumor-infiltrating immune cells between high and low GNPAT expression groups. **(C)** IPS analysis predicting response to CTLA-4 and/or PD-1 blockade therapy. **(D)** Analysis of classic immune checkpoint molecule expression (CD274, CTLA4, PDCD1, PDCD1LG2). **(E)** TIDE score analysis predicting response to immunotherapy. **(F)** Drug sensitivity analysis (IC50 values) for various therapeutic agents. **p < 0.01.

Stratification based on median GNPAT expression revealed significant alterations in the TME. While tumors with high GNPAT expression exhibited elevated immune scores (**Figure 2A**), further analysis indicated that this was driven by a distinct leukocyte composition, characterized by significant enrichment of immunosuppressive cell types, particularly M0 macrophages and neutrophils (**Figure 2B**). This pattern of infiltration is associated with impaired anti-tumor immunity, which aligns with the significantly higher TIDE scores observed in the high-GNPAT group (**Figure 2E**), predicting greater immune evasion and poorer response to immunotherapy. Analysis of immunophenoscore suggested a possibility of response to combined PD-1/CTLA-4 blockade in specific subsets (**Figure 2C**), although no significant difference was found in the expression of conventional immune checkpoint molecules (**Figure 2D**). Consistent with an altered metabolic state, pharmacogenomic analysis predicted increased sensitivity to several therapeutic agents, including Sorafenib, Cyclopamine, Embelin, PAC-1, and AKT inhibitor VIII, in high-GNPAT tumors (**Figure 2F**).

### 3.3 Experimental validation confirms GNPAT overexpression and reveals peroxisomal reprogramming

**Figure 3:**
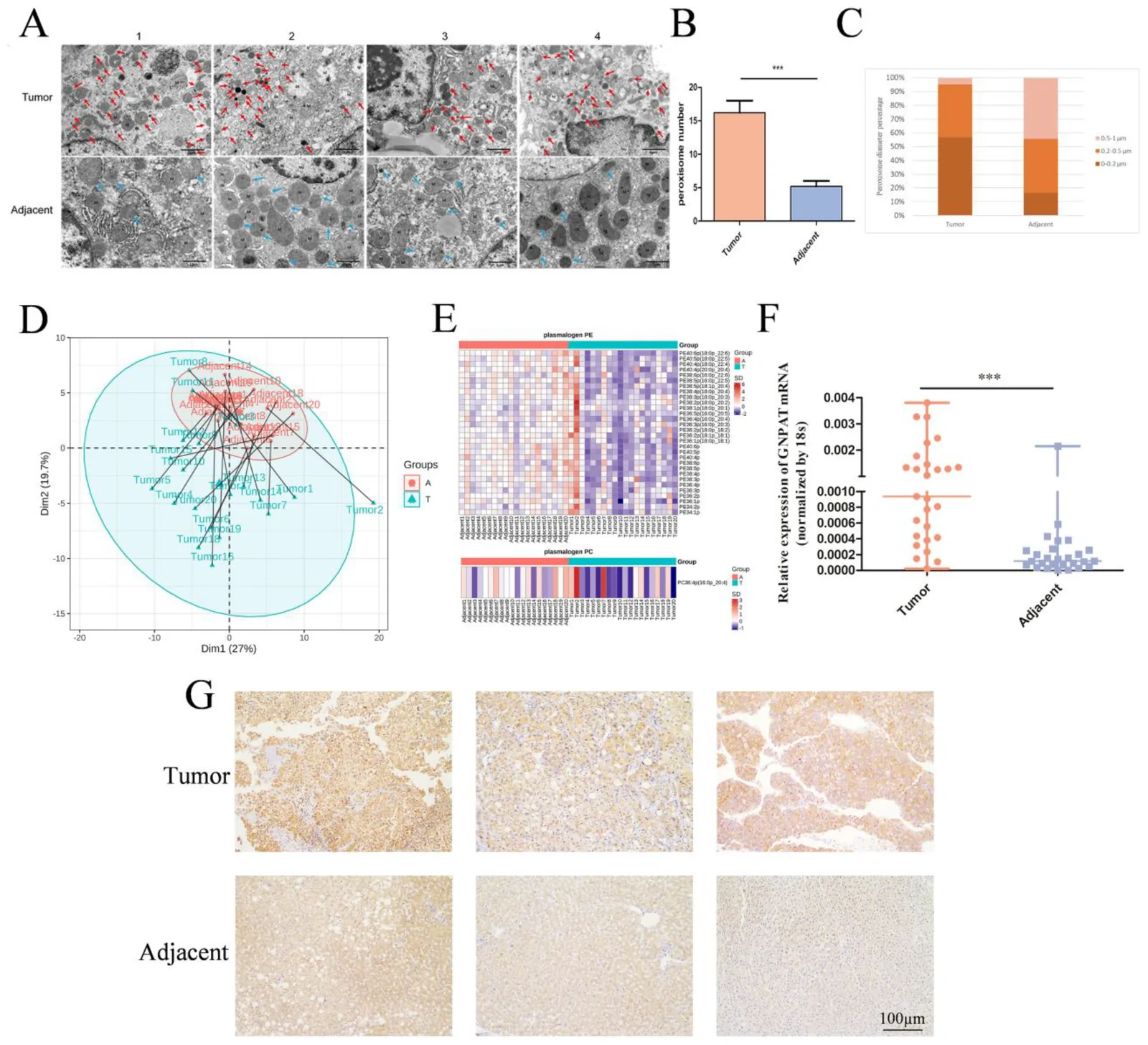
Experimental validation confirms GNPAT overexpression and reveals peroxisomal alterations and lipidomic reprogramming in HCC. **(A)** Transmission electron microscopy (TEM) images of peroxisomes (indicated by arrows) in adjacent and tumor tissues. (Scale bar, 5 μm). **(B)** Quantitative analysis of the number of peroxisomes per field from TEM images. **(C)** Percentage distribution of peroxisomes with different diameters in tumor vs. adjacent tissues. **(D)** PCA score plot of lipidomic data from 20 pairs of HCC and adjacent tissues. **(E)** Heatmap showing the relative levels of various pPE and pPC species in tumors (T) and adjacent tissues (A). **(F)** RT-qPCR analysis of GNPAT mRNA expression levels in HCC and adjacent tissues. **(G)** Immunohistochemical (IHC) staining images and quantitative analysis of GNPAT protein expression in HCC and adjacent non-tumor tissues. ***p < 0.001.

Clinical specimen multimodal validation showed there were significant peroxisomal changes in HCC. The transmission electron microscopy showed a higher density of the peroxisomes with fewer organellar diameters in tumor tissues (**Figures 3A–C**). Lipidomic profiling of 20 matched pairs revealed a complex alteration in plasmalogen species. Most plasmalogen species, including both plasmenylethanolamine (pPE) and plasmenylcholine (pPC) subtypes, showed an overall decrease in tumor tissues (**Figures 3D, E**). This suggests a general reduction rather than a selective remodeling of the plasmalogen pool in HCC. The expression of GNPAT in malignant tissues was always corroborated by transcriptional and protein analysis (**Figures 3F, G**).

### 3.4 GNPAT regulates plasmalogen biosynthesis and peroxisomal homeostasis

**Figure 4:**
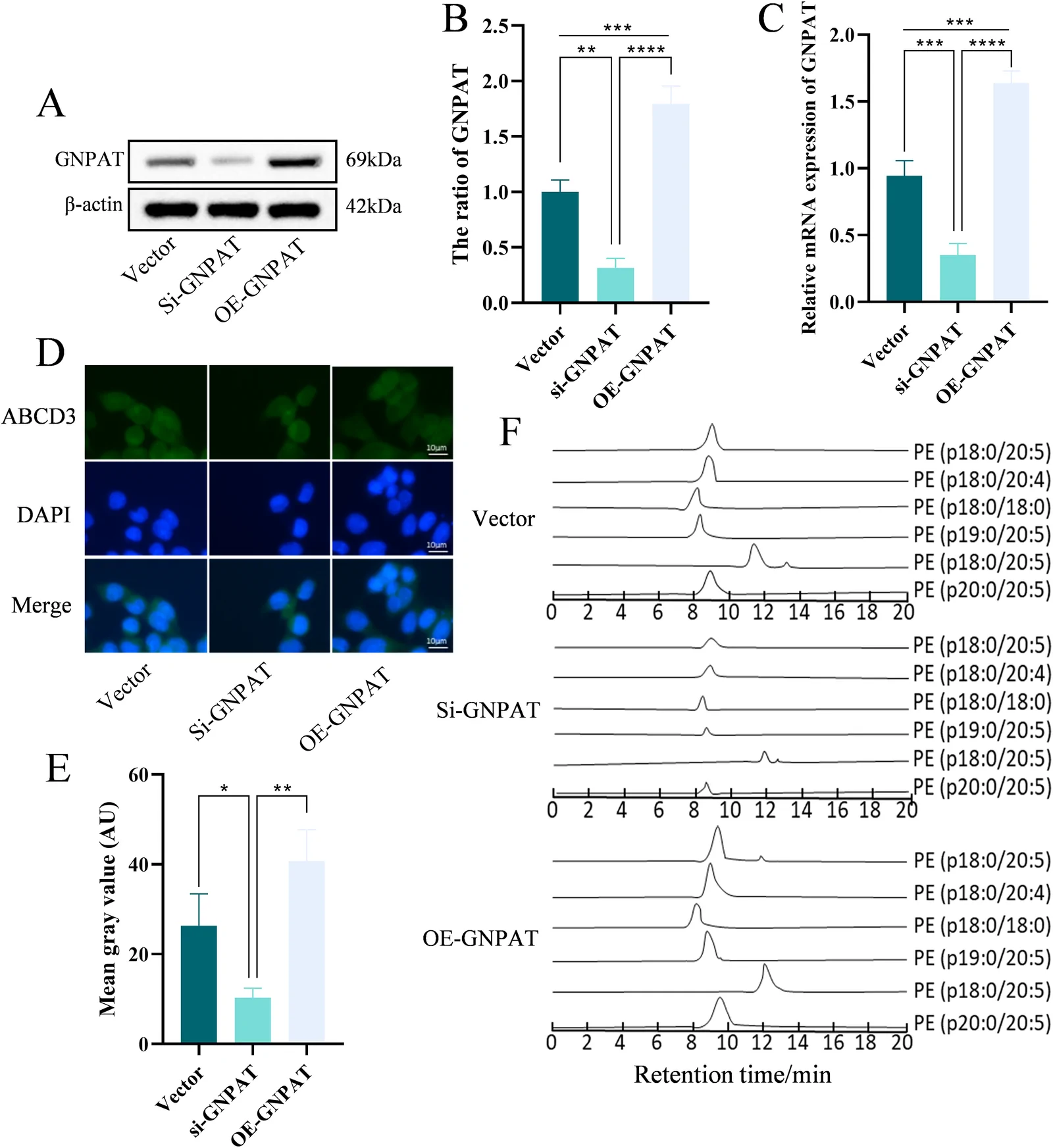
GNPAT regulates peroxisomal function and plasmalogen synthesis. **(A, B)** Western blot analysis of GNPAT protein expression (n=3). **(C)** RT-qPCR analysis of GNPAT mRNA expression in transfected HepG2 cells (n=6). **(D, E)** Representative immunofluorescence images (top) and quantitative analysis (bottom) of peroxisomes labeled with ABCD3 antibody. Scale bar, 10 μm. **(F)** Cellular pPE levels detected by LC-MS/MS. *p < 0.05, **p < 0.01, ***p < 0.001, ****p < 0.0001.

Efficient GNPAT modulation in HepG2 cells was confirmed through transcriptional and protein assessment (**Figures 4A–C**). Immunofluorescence analysis demonstrated that GNPAT overexpression enhanced peroxisomal abundance while knockdown produced opposite effects (**Figures 4D, E**). LC-MS/MS quantification revealed that GNPAT elevation significantly increased pPE levels (**Figure 4F**), supporting a central role for GNPAT in regulating plasmalogen biosynthesis.

### 3.5 Plasmalogen-activated PPARγ signaling drives HCC malignant progression

**Figure 5:**
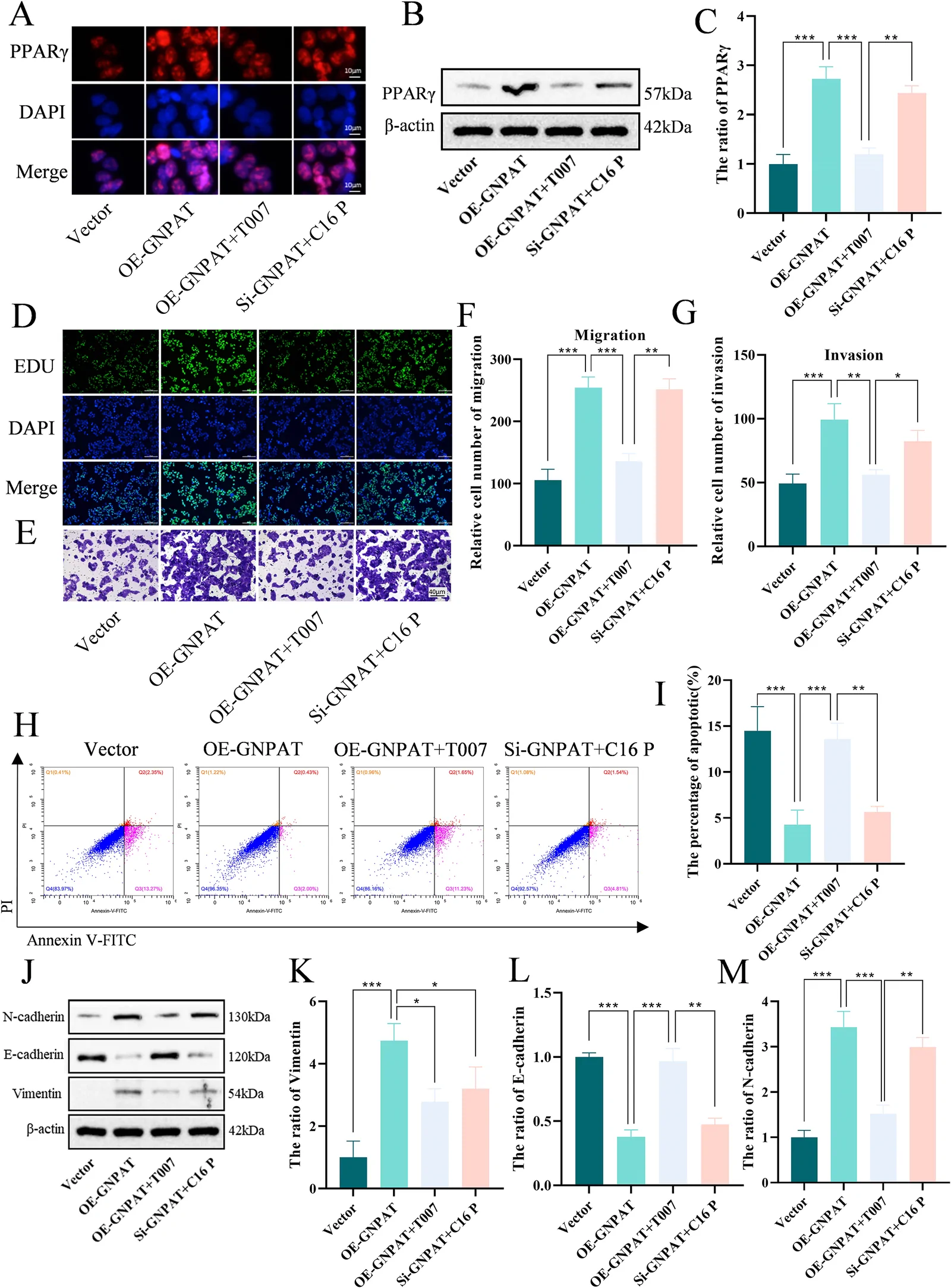
Plasmalogens promote malignant progression of HCC via activation of the PPARγ pathway. **(A)** Representative immunofluorescence images showing the subcellular localization of PPARγ (red). Nuclei were counterstained with DAPI (blue). Scale bar, 10 μm. **(B, C)** Western blot analysis of PPARγ protein expression (n=3). **(D)** Cell proliferation assessed by EdU assay. Scale bar, 100 μm. **(E-G)** Cell migration and invasion assessed by Transwell assays. Scale bar, 40 μm. **(H, I)** Cell apoptosis evaluated by flow cytometry (Annexin V/PI staining) (n=3). **(J-M)** Western blot analysis of EMT marker protein expression (n=3). *p < 0.05, **p < 0.01, ***p < 0.001.

Mechanistic investigation revealed PPARγ pathway activation upon GNPAT overexpression, characterized by enhanced protein expression and increased nuclear accumulation of PPARγ (**Figures 5A–C**). This observed nuclear accumulation is consistent with pathway activation and is further corroborated by the functional rescue upon pharmacological inhibition of PPARγ. Functional assays demonstrated that GNPAT potentiated proliferation, migration, epithelial-mesenchymal transition, and apoptosis resistance (**Figures 5D–M**). Specifically, GNPAT overexpression significantly enhanced cell proliferation (**Figure 5D**), promoted migratory and invasive capacities (**Figures 5E–G**), and induced a shift towards a mesenchymal phenotype, as evidenced by decreased E-cadherin and increased N-cadherin/Vimentin expression (**Figures 5J–M**). Conversely, GNPAT knockdown or PPARγ inhibition with T0070907 consistently reversed these pro-tumorigenic phenotypes, confirming the dependency of these processes on the GNPAT-PPARγ axis.

### 3.6 GNPAT-mediated plasmalogen production promotes M2 macrophage polarization

**Figure 6:**
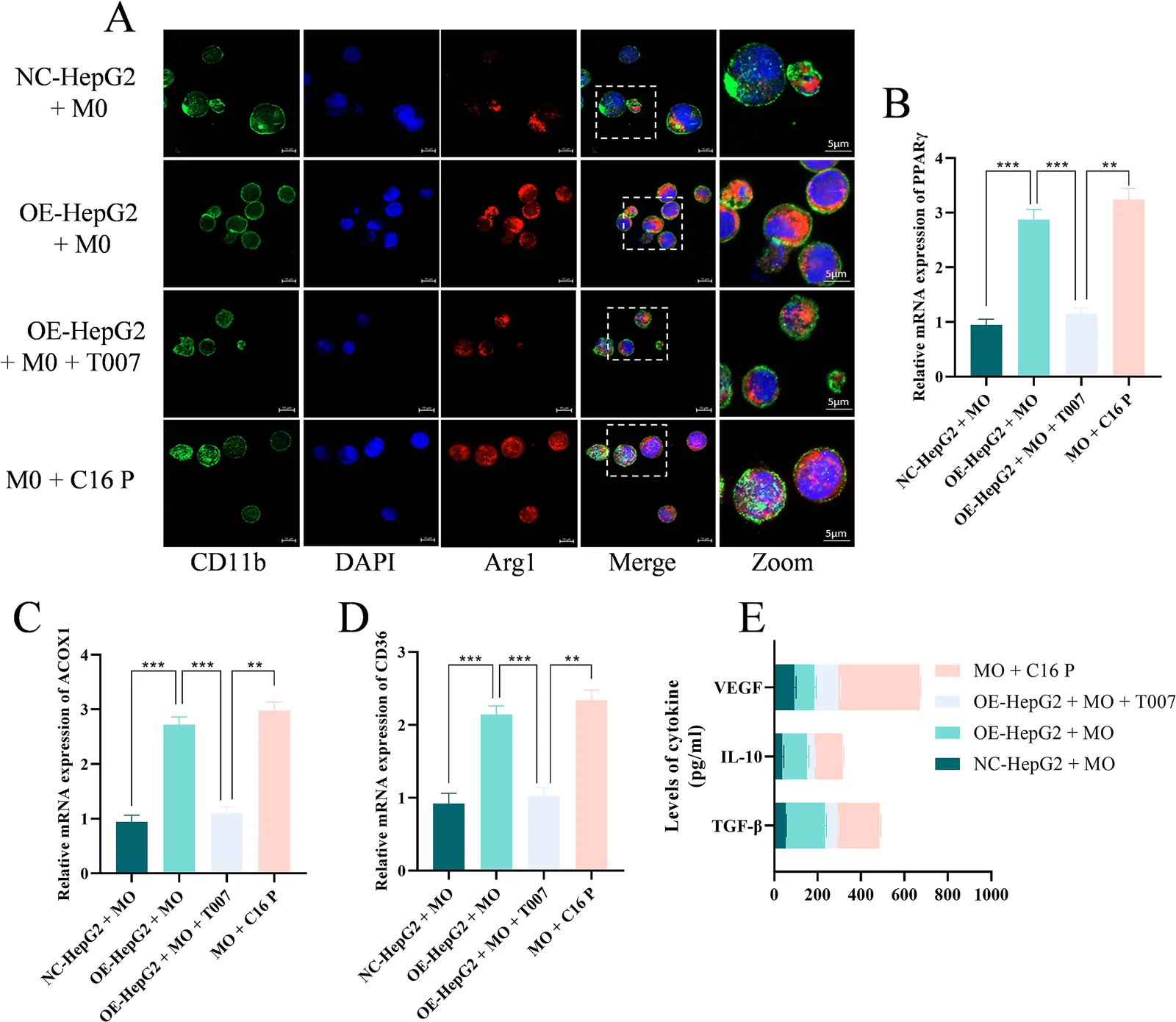
GNPAT induces macrophage M2 polarization via plasmalogens. **(A)** Representative immunofluorescence images showing the expression of Arg1 (red, M2 marker) and CD11b (green, macrophage marker) in macrophages. Scale bar, 10 μm. **(B-D)** RT-qPCR analysis of PPARγ and its target genes ACOX1 and CD36 mRNA expression in macrophages (n=6). **(E)** ELISA detection of the secretion levels of immunosuppressive factors (TGF-β, IL-10) and pro-angiogenic factor (VEGF) in the co-culture supernatant (n=6). **p < 0.01, ***p < 0.001.

To investigate the immunomodulatory effects of GNPAT in the tumor immune microenvironment, a co-culture system was established that would include cells of HCC and THP-1-differentiated macrophages. The immunofluorescence analysis revealed substantial upregulation of arginase-1 (Arg1) which is one of the canonical M2 polarization markers in macrophages in co-culture with GNPAT-overexpressing HepG2 cells relative to the control conditions (**Figure 6A**). Analysis of PPARγ signaling revealed that macrophages upon observation of high-GNPAT HCC cells exhibited increased transcriptional levels of PPARγ and its downstream metabolic targets ACOX1 and CD36 (**Figures 6B–D**). Direct administration of C16:0 plasmalogen to macrophages was sufficient to induce PPARγ pathway activation and M2 polarization, supporting the role of plasmalogens as key signaling mediators in this cross-talk. Cytokine profiling of the co-culture supernatants revealed increased release of immunosuppressive factors (TGF-β, IL-10) and the angiogenic mediator VEGF (**Figure 6E**). Stunningly, all the above effects were inhibited by the PPARγ antagonist T0070907 and demonstrated that PPARγ is the primary regulator of this immunometabolic axis.

## 4 Discussion

The epidemiology of HCC has undergone a dramatic change with metabolic dysfunction related steatotic liver disease (MASLD) now being the major etiology in many geographical regions replacing that of viral hepatitis. Such an epidemiological transition has been strongly linked to an increasing trend in obesity and established MASLD as the fastest-growing etiology leading to liver transplantation for HCC (1, 2, 29–31). This transition, linked to rising obesity rates, positions MASLD as the fastest-growing indication for liver transplantation in HCC. Concurrently, metabolic reprogramming is recognized as central to HCC pathogenesis, with peroxisomes emerging as critical organelles in lipid metabolism (32). GNPAT was identified in the present study as a key peroxisomal enzyme that serves as a molecular nexus linking peroxisomal lipid metabolism to malignant progression and immunosuppression, mechanistically through plasmalogen-mediated activation of PPARγ.

The high expression of GNPAT in HCC specimens is congruent with reported peroxisomal defects in hepatic malignancy (25). GNPAT was found to be to be highly expressed in HCC specimens. Its identification via machine learning and validation as an independent prognostic factor robustly supports its utility as a prognostic biomarker. Functional annotation revealed GNPAT’s primary involvement in peroxisomal pathways and ether lipid biosynthesis. Notably, high GNPAT expression was paradoxically associated with both elevated immune scores and poorer patient survival. This apparent contradiction is resolved by analyzing the qualitative nature of the immune infiltrate. The elevated immune score in high-GNPAT tumors is driven predominantly by an increase in immunosuppressive cell populations, particularly M0 macrophages and neutrophils, rather than anti-tumor effector cells. This interpretation is further bolstered by the concurrently higher TIDE scores in this cohort, which computationally predict a dysfunctional and evasion-prone tumor immune microenvironment. Consequently, GNPAT expression correlates with the establishment of a qualitatively immunosuppressive and therapy-resistant niche, explaining its strong association with aggressive disease and predicting reduced responsiveness to immune checkpoint inhibitors. These findings resonate with the emerging theme linking lipid metabolic rewiring to immunotherapy resistance, positioning GNPAT as a compelling therapeutic target to overcome immune evasion.

Lipidomic profiling of clinical tissues revealed an overall reduction in the plasmalogen pool within HCC tumors (**Figures 3D, E**), consistent with a state of generalized peroxisomal dysfunction in advanced malignancies. This suggests a plasmalogen-deficient landscape in the bulk HCC microenvironment. Intriguingly, and seemingly paradoxically, our functional studies demonstrated that GNPAT overexpression robustly enhances intracellular plasmalogen (pPE) levels (**Figure 4F**), activates oncogenic PPARγ signaling, and drives pro-tumorigenic phenotypes. To reconcile these observations, a model of metabolic adaptation and niche construction is proposed. Within the globally plasmalogen-depleted tumor ecosystem, a subset of HCC cells with high GNPAT expression acquires a metabolic advantage by autonomously sustaining or elevating plasmalogen synthesis. This endogenous production fuels PPARγ-mediated survival and growth signals in the tumor cells themselves. Furthermore, through the secretion of these peroxisome-derived lipids, these GNPAT-high cells can actively remodel their microenvironment, promoting M2-like macrophage polarization and establishing an immunosuppressive niche conducive to tumor progression. Thus, GNPAT functions not as a passive biomarker of peroxisomal activity, but as a critical metabolic adaptor, enabling aggressive tumor cell subsets to thrive amidst—and even exploit—the broader metabolic alterations of the TME. This model underscores GNPAT’s potential as a therapeutic target, as its inhibition could selectively disrupt this adaptive pathway in treatment-resistant cell populations.

Mechanistically, this study demonstrated that GNPAT enhances plasmalogen production, which in turn promotes PPARγ signaling activation, driving HCC cell proliferation, migration, invasion, and resistance to apoptosis. Crucially, GNPAT-overexpressing HCC cells promoted M2-like macrophage polarization via plasmalogen secretion, leading to increased production of immunosuppressive (TGF-β, IL-10) and pro-angiogenic (VEGF) factors. The complete reversal of these phenotypes upon pharmacological inhibition of PPARγ validates the specificity of the GNPAT-plasmalogen-PPARγ axis in orchestrating this immunometabolic crosstalk.

There are several limitations that need to be noted. First, the molecular nature of the peroxisomal dysregulation brought about by GNPAT has not been fully characterized. Second, the prognostic utility of GNPAT remains to be validated in larger, independent cohorts. Third, while our immunofluorescence data indicate enhanced nuclear accumulation of PPARγ upon GNPAT overexpression, future studies employing time-course experiments would be valuable to delineate the precise kinetics of PPARγ nuclear translocation and activation in this context. Fourth, although our data support a model of plasmalogen-mediated PPARγ pathway activation, further mechanistic studies (e.g., direct binding assays) are required to definitively establish plasmalogens as endogenous ligands of PPARγ. Fifth, the therapeutic practicality of targeting peroxisomal lipid metabolism *in vivo* requires further investigation.

The GNPAT-plasmalogen-PPARγ axis is mechanistically defined as a novel regulator of immunometabolic crosstalk in HCC, providing a strong preclinical rationale for targeting this pathway. To advance this finding, the essential next step is validation in immunocompetent animal models to assess whether inhibiting this axis can remodel the TME and synergize with therapies. Future research should focus on several key directions: developing selective GNPAT inhibitors and evaluating their combination with immunotherapies; experimentally validating the predicted drug sensitivities (e.g., to Sorafenib) and elucidating the underlying mechanisms linking this axis to drug response; employing comprehensive macrophage polarization markers (including both M1 and M2 signatures) to clarify the immunomodulatory impact; and exploring whether plasmalogens signal through alternative receptors (e.g., GPCRs, TLRs) or pathways beyond PPARγ.

## 5 Conclusion

GNPAT was identified as a central peroxisomal enzyme that integrates simultaneous metabolic and immunological reprogramming in HCC. GNPAT is mechanistically connected to the plasmalogen biosynthesis in order to stimulate the activity of PPARγ and thus progresses malignant events in HCC cells and induces M2-like macrophage polarization. The association between high GNPAT expression and an immunosuppressive microenvironment, along with predicted poor response to immune checkpoint blockade, suggests its potential as a therapeutic target. However, definitive translational relevance requires further validation *in vivo*. The GNPAT-plasmalogen-PPARγ axis represents a promising preclinical target; future research should prioritize developing and testing selective inhibitors in animal models to evaluate their potential for overcoming immunosuppression and improving therapeutic outcomes.

## Data Availability

The datasets presented in this article are not readily available because of ethical and privacy restrictions. Requests to access the datasets should be directed to the corresponding author/s.
