# Correction: GNPAT promotes immunosuppression in hepatocellular carcinoma by activating the plasmalogen-PPARg pathway to drive M2 macrophage polarization

**DOI:** 10.3389/fimmu.2026.1901435

**Published:** 2026-07-02

**Authors:** Meng Hu, Nan Zhang, Ya-Qi Wang, Xiao-Ming Wang, Yun Shi, Min Yao, Lian-Guo Hou, Ling-Ling Jiang

**Affiliations:** 1 Ministry of Education Key Laboratory of Neural and Vascular Biologys, Department of Biochemistry and Molecular Biology, Hebei Medical University, Shijiazhuang, Hebei, China; 2 Department of Complex Preparation, Shijiazhuang No. 4 Pharmaceutical, Shijiazhuang, Hebei, China; 3 College of Integrative Chinese and Western Medicine, Hebei University of Chinese Medicine, Shijiazhuang, Hebei, China; 4 Department of Clinical Laboratory, Hebei Province Hospital of Chinese Medicine, Shijiazhuang, Hebei, China; 5 Department of Clinical Laboratory, The First Hospital of Tsinghua University, Beijing, China

**Keywords:** biomarkers, drug sensitivity prediction, GNPAT, hepatocellular carcinoma, immune cell infiltration, peroxisome

There was a mistake in **Figure 3** as published. In **Figure 3E**, “PE” should be replaced by “plasmalogen PE”. The corrected **Figure 3** appears below.

**Figure 3:**
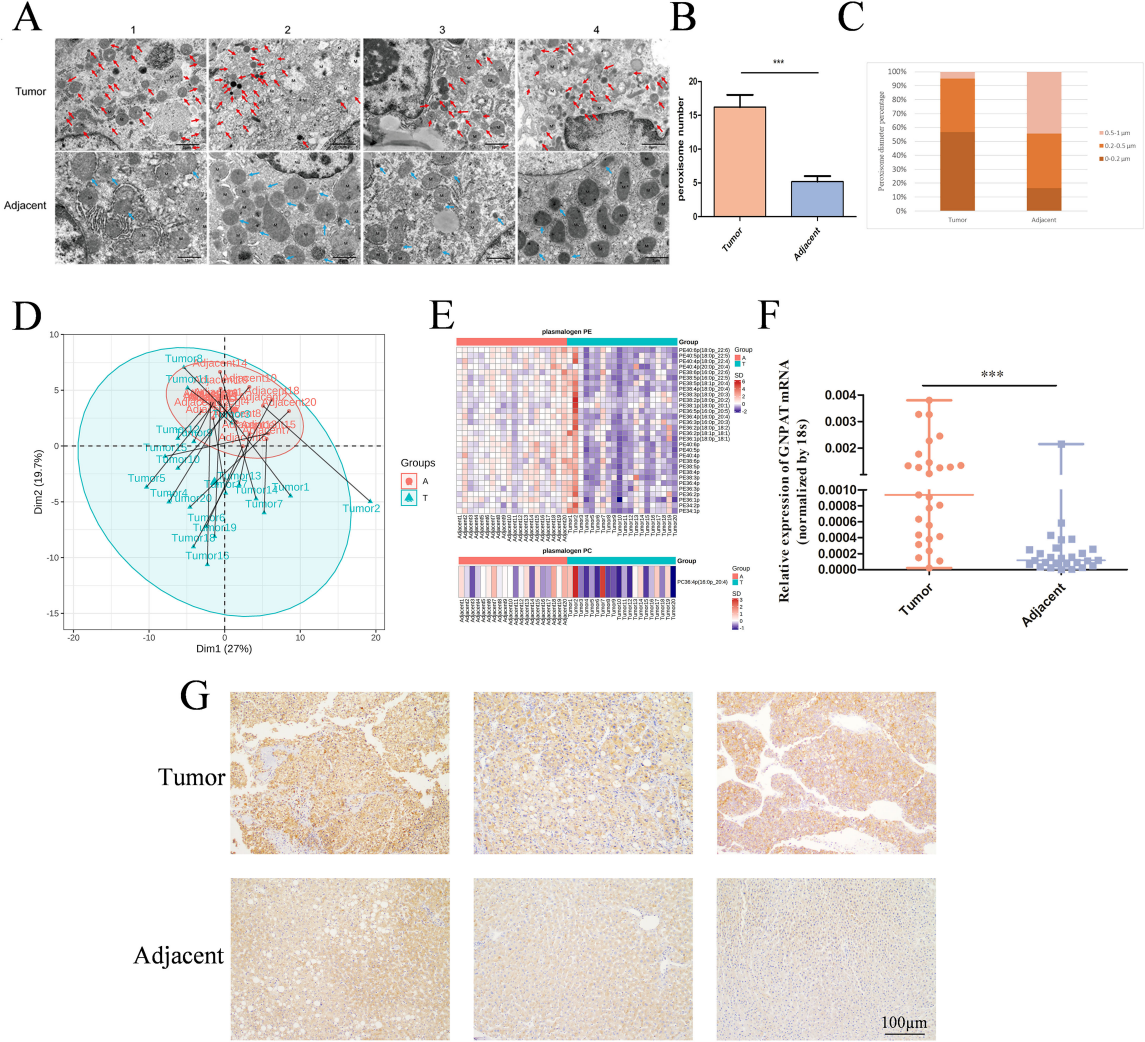
Experimental validation confirms GNPAT overexpression and reveals peroxisomal alterations and lipidomic reprogramming in HCC. **(A)** Transmission electron microscopy (TEM) images of peroxisomes (indicated by arrows) in adjacent and tumor tissues. (Scale bar, 5 µm). **(B)** Quantitative analysis of the number of peroxisomes per field from TEM images. **(C)** Percentage distribution of peroxisomes with different diameters in tumor vs. adjacent tissues. **(D)** PCA score plot of lipidomic data from 20 pairs of HCC and adjacent tissues. **(E)** Heatmap showing the relative levels of various pPE and pPC species in tumors (T) and adjacent tissues (A). **(F)** RT-qPCR analysis of GNPAT mRNA expression levels in HCC and adjacent tissues. **(G)** Immunohistochemical (IHC) staining images and quantitative analysis of GNPAT protein expression in HCC and adjacent non-tumor tissues. ***p < 0.001.

Furthermore, there was a mistake in the caption of **Figure 3** as published. “(A, F)” should be replaced by “(A). (F)”. The corrected caption of **Figure 3** appears below.

“**Figure 3** Experimental validation confirms GNPAT overexpression and reveals peroxisomal alterations and lipidomic reprogramming in HCC. (A) Transmission electron microscopy (TEM) images of peroxisomes (indicated by arrows) in adjacent and tumor tissues. (Scale bar, 5 µm). (B) Quantitative analysis of the number of peroxisomes per field from TEM images. (C) Percentage distribution of peroxisomes with different diameters in tumor vs. adjacent tissues. (D) PCA score plot of lipidomic data from 20 pairs of HCC and adjacent tissues. (E) Heatmap showing the relative levels of various pPE and pPC species in tumors (T) and adjacent tissues (A). (F) RT-qPCR analysis of GNPAT mRNA expression levels in HCC and adjacent tissues. (G) Immunohistochemical (IHC) staining images and quantitative analysis of GNPAT protein expression in HCC and adjacent non-tumor tissues. ***p < 0.001.”

The original version of this article has been updated.

