# Correction: GNPAT promotes immunosuppression in hepatocellular carcinoma by activating the plasmalogen-PPARγ pathway to drive M2 macrophage polarization

**DOI:** 10.3389/fimmu.2026.2005722

**Published:** 2026-10-19

**Authors:** Meng Hu, Nan Zhang, Ya-Qi Wang, Xiao-Ming Wang, Yun Shi, Min Yao, Lian-Guo Hou, Ling-Ling Jiang

**Affiliations:** 1 Ministry of Education Key Laboratory of Neural and Vascular Biologys, Department of Biochemistry and Molecular Biology, Hebei Medical University, Shijiazhuang, Hebei, China; 2 Department of Complex Preparation, Shijiazhuang No. 4 Pharmaceutical, Shijiazhuang, Hebei, China; 3 College of Integrative Chinese and Western Medicine, Hebei University of Chinese Medicine, Shijiazhuang, Hebei, China; 4 Department of Clinical Laboratory, Hebei Province Hospital of Chinese Medicine, Shijiazhuang, Hebei, China; 5 Department of Clinical Laboratory, The First Hospital of Tsinghua University, Beijing, China

**Keywords:** biomarkers, drug sensitivity prediction, GNPAT, hepatocellular carcinoma, immune cell infiltration, peroxisome

There was a mistake in **Figure 4** as published. In **Figure 4D**, two fluorescence images were inadvertently inserted incorrectly during figure assembly. This error occurred during the selection and arrangement of representative images and did not involve the underlying experimental data. We have now replaced the two incorrect images with the corresponding correct representative images from the original experimental dataset. The quantitative analyses, statistical results, and conclusions of the article remain unchanged.

The authors apologize for this error and state that this does not change the scientific conclusions of the article in any way.

The corrected **Figure 4** appears below.

**Figure 4:**
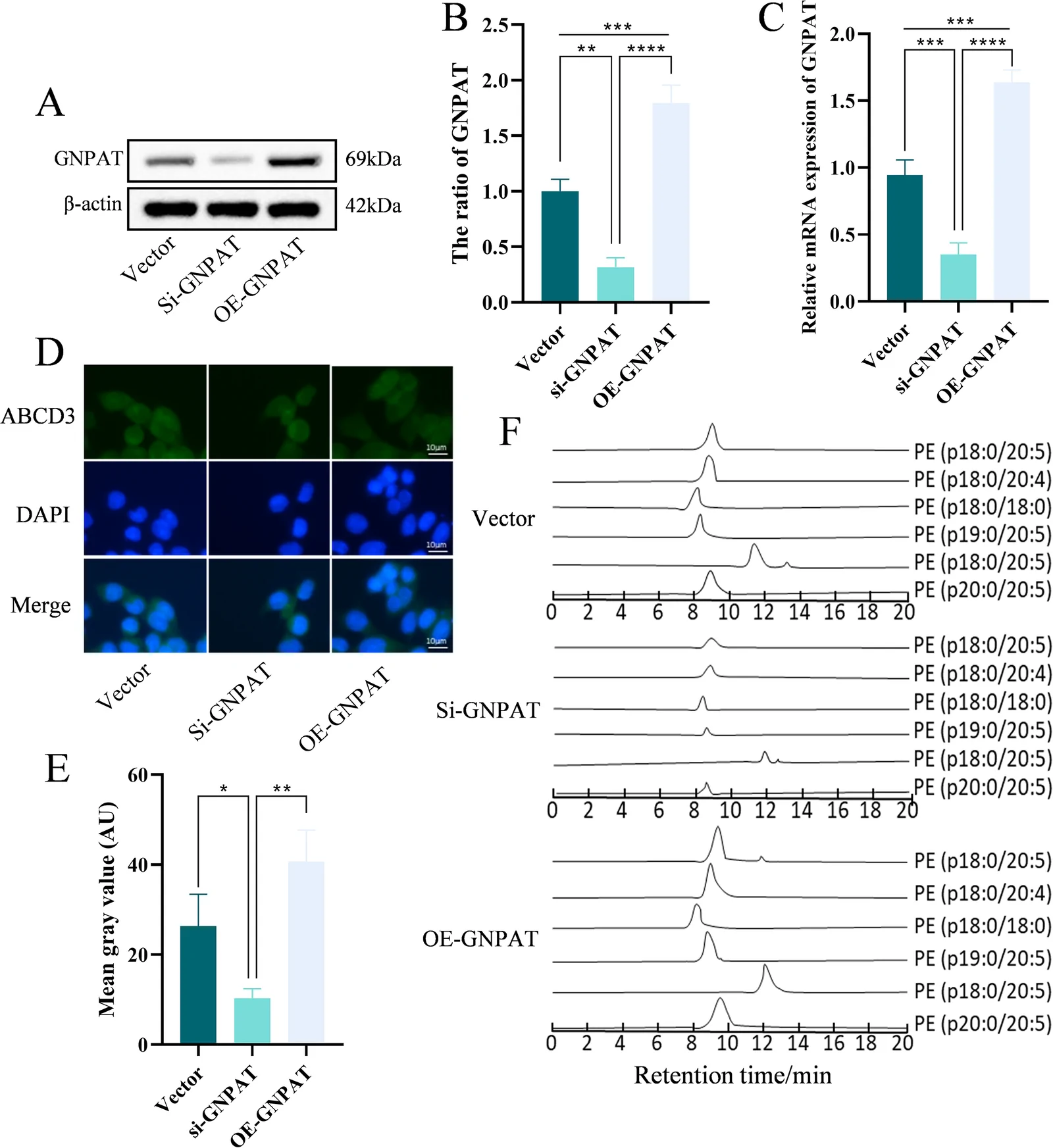
GNPAT regulates peroxisomal function and plasmalogen synthesis. **(A, B)** Western blot analysis of GNPAT protein expression (n=3). **(C)** RT-qPCR analysis of GNPAT mRNA expression in transfected HepG2 cells (n=6). **(D, E)** Representative immunofluorescence images (top) and quantitative analysis (bottom) of peroxisomes labeled with ABCD3 antibody. Scale bar, 10 μm. **(F)** Cellular pPE levels detected by LC-MS/MS. *p < 0.05, **p < 0.01, ***p < 0.001, ****p < 0.0001.

The original article has been updated.

